# Effects of Proportions of Dietary Macronutrients on Glucocorticoid Metabolism in Diet-Induced Obesity in Rats

**DOI:** 10.1371/journal.pone.0008779

**Published:** 2010-01-19

**Authors:** Roland H. Stimson, Gerald E. Lobley, Ioanna Maraki, Nicholas M. Morton, Ruth Andrew, Brian R. Walker

**Affiliations:** 1 Centre for Cardiovascular Sciences, Queen's Medical Research Institute, University of Edinburgh, Edinburgh, United Kingdom; 2 Division of Obesity and Metabolic Health, Rowett Institute of Nutrition and Health, University of Aberdeen, Aberdeen, United Kingdom; University of Camerino, Italy

## Abstract

Tissue glucocorticoid levels in the liver and adipose tissue are regulated by regeneration of inactive glucocorticoid by 11β-hydroxysteroid dehydrogenase type 1 (11β-HSD1) and inactivation by 5α- and 5β-reductases. A low carbohydrate diet increases hepatic 11β-HSD1 and reduces glucocorticoid metabolism during weight loss in obese humans. We hypothesized that similar variations in macronutrient proportions regulate glucocorticoid metabolism in obese rats. Male Lister Hooded rats were fed an obesity-inducing ad libitum ‘Western’ diet (37% fat, n = 36) for 22 weeks, then randomised to continue this diet (n = 12) or to switch to either a low carbohydrate (n = 12) or a moderate carbohydrate (n = 12) diet for the final 8 weeks. A parallel lean control group were fed an ad libitum control diet (10% fat, n = 12) throughout. The low and moderate carbohydrate diets decreased hepatic 11β-HSD1 mRNA compared with the Western diet (both 0.7±0.0 vs 0.9±0.1 AU; p<0.01), but did not alter 11β-HSD1 in adipose tissue. 5α-Reductase mRNA was increased on the low carbohydrate compared with the moderate carbohydrate diet. Compared with lean controls, the Western diet decreased 11β-HSD1 activity (1.6±0.1 vs 2.8±0.1 nmol/mcg protein/hr; p<0.001) and increased 5α-reductase and 5β-reductase mRNAs (1.9±0.3 vs 1.0±0.2 and 1.6±0.1 vs 1.0±0.1 AU respectively; p<0.01) in the liver, and reduced 11β-HSD1 mRNA and activity (both p<0.01) in adipose tissue. Although an obesity-inducing high fat diet in rats recapitulates the abnormal glucocorticoid metabolism associated with human obesity in liver (but not in adipose tissue), a low carbohydrate diet does not increase hepatic 11β-HSD1 in obese rats as occurs in humans.

## Introduction

Glucocorticoids are potent regulators of energy metabolism. In addition to production by the adrenal glands, tissue glucocorticoid levels are regulated by several enzymes, particularly 11β-hydroxysteroid dehydrogenase type 1 (11β-HSD1). 11β-HSD1 functions in vivo to regenerate cortisol from inactive cortisone (corticosterone from inactive 11-dehydrocorticosterone in rodents) and is highly expressed in the liver and adipose tissue [1]. Glucocorticoids are metabolised primarily by 5α-reductase type 1 and 5β-reductase in the liver prior to further metabolism by 3α-hydroxysteroid dehydrogenase, while 5α-reductase type 1 is also present in adipose tissue [2]. Control of tissue glucocorticoids is dysregulated in human obesity, with decreased cortisol levels in the liver secondary to reduced 11β-HSD1 activity [3], [4] and increased 5α- and 5β-reductase activity [5], [6]. Conversely, 11β-HSD1 mRNA and activity are increased in adipose tissue [4], [7]–[9], amplifying tissue glucocorticoid levels. Similarly, in obese Zucker rats, 5α- and 5β-reductase activities are increased and 11β-HSD1 is decreased in the liver, while 11β-HSD1 is increased in adipose tissue [10], [11]. The mechanism of these tissue-specific changes in glucocorticoid metabolism is unknown, but may reflect abnormal dietary intake in obesity.

Dietary macronutrient content is an important determinant of metabolic health independent of changes in body weight [12], [13], and this may be partly mediated by changes in glucocorticoid action. Glucocorticoids respond acutely to changes in nutritional status, for example plasma cortisol concentrations rise within minutes of eating [14], while tissue glucocorticoids are regulated more chronically by dietary content. A diet-induced obesity model using an ad libitum high fat diet (45% of total calories) for 3 weeks in rats recapitulated the abnormal hepatic glucocorticoid metabolism observed in human obesity, with decreased 11β-HSD1 and increased hepatic 5β-reductase activities, although this effect was not maintained after 20 weeks [15]. However, in contrast to human obesity, 11β-HSD1 activity was decreased in adipose tissue on the high fat diet [15]. Similarly, mice fed a high fat diet (58% fat) ad libitum for 2 or 18 weeks dramatically decreased 11β-HSD1 mRNA and activity in subcutaneous, visceral and epididymal adipose tissue but had no effect on hepatic 11β-HSD1 [16].

In humans, several studies have examined the effect of underfeeding on 11β-HSD1 but have found either increased [17], unchanged [18], or decreased 11β-HSD1 [19] in subcutaneous adipose tissue after weight loss, while hepatic 11β-HSD1 activity was unaltered [17]. This inconsistency may be attributable to the various dietary strategies used in these studies. We recently examined the effects of dietary composition during weight loss in obese humans, and found that a high fat-low carbohydrate (low CHO) diet increased whole body (predominantly hepatic) 11β-HSD1 activity and decreased 5α- and 5β-reductase activities (measured using deuterated cortisol infusions) compared with a moderate fat-moderate carbohydrate (moderate CHO) diet, with no effect on subcutaneous adipose tissue 11β-HSD1 [20]. Furthermore, dietary regulation of 11β-HSD1 was independent of changes in body weight.

The discordant results between human and rodent studies could be due to differences in regulation of enzyme expression between species, but the macronutrient content of the diets used in the rodent studies differ markedly from those achievable in human diets. In order to test whether dietary regulation of glucocorticoid metabolism is similar between species, and potentially to establish a model in which to dissect the mediators of the effects of diet, we fed rats a human-equivalent ‘Western’ diet to induce obesity in order to mimic the subjects who enrolled in our human study. We then followed their responses to diets with similar macronutrient content as the low CHO and moderate CHO diets previously used in humans [20], and explored glucocorticoid metabolism in the liver and adipose tissue.

## Methods

### Ethics Statement

All procedures were approved by the Animal Ethics Committee of the Rowett Institute of Nutrition and Health (RINH), under the UK Animals (Scientific Procedures) Act 1996.

### Animals and Protocol

Details of the protocol have been described previously [21]. 48 male Lister Hooded rats were weaned at 19 days of age onto a stock (control) diet (CRM (pellets), Special Diet Services, Essex, UK). Caloric content of this diet was carbohydrate (CHO) 69%, protein 22%, fat 9%. After 9 days on this diet (t = 0 weeks), the rats were randomised to two groups: a lean control group (n = 12), fed the control diet ad libitum for 30 weeks; and a diet-induced obesity group (n = 36), fed an ad libitum high fat ‘Western’ diet for 22 weeks (CHO 47%, protein 16%, fat 37%; prepared in-house at the Rowett Institute of Nutrition and Health) [21]. After 22 weeks, obese rats were randomised to 3 further sub-groups (n = 12 each): one group remained on the Western diet; a second was placed on a ketogenic high fat-low carbohydrate (low CHO) diet (CHO 7%, protein 37%, fat 56%); while a third was commenced on a moderate fat-moderate carbohydrate (moderate CHO) diet (CHO 27%, protein 37%, fat 36%). The Western and moderate CHO diets were iso-energetic while the low CHO was more energy dense, due to the additional fat. The exact content of these diets has been published in detail previously [21]. Casein was used for the dietary protein. Maize oil was used for baseline fat content of each in-house diet, with suet used to make up the additional fat on the low CHO diet. Cornstarch was the main CHO source for each diet with sucrose added to the ‘Western’ diet. All three sub-groups were fed ad libitum for a further 8 weeks.

Rats were housed four to a cage for the first 18 weeks then singly for the remaining 12 weeks, in a 12: 12 hour light: dark cycle (0700–1900 h light). Energy intake was recorded daily by measuring food intake per cage and the rats were weighed twice weekly. In a sub-set of rats within each group 4-day collections of faeces were used to estimate the apparent digestibility of the various diets. Body composition was determined at 22 and 30 weeks using a MRI scanner (EchoMRI, Houston, Texas, USA). Energy expenditure was estimated indirectly from incremental digestible energy intake, minus an estimated urine energy loss, and calculated changes in body energy deposition (based on fat and lean composition). An oral glucose tolerance test (OGTT) was performed after 28 weeks at 8am after overnight fasting, using gavage of a 50% glucose solution equivalent to 2 g glucose/kg body weight. Blood samples were taken by tail tip prior to gavage then at 7, 15, 30, 60, 90 and 120 minutes. After 30 weeks, the rats were anaesthetised and decapitated in the fed state at 1000 h. Organs were immediately removed, including the liver and peri-renal adipose tissue, snap-frozen on dry ice and stored at −80°C until analysis.

### Laboratory Measurements

Plasma insulin and glucose were analysed as described previously [21]. Liver and peri-renal adipose tissue were analysed for 11β-HSD1, 5α-reductase type 1, 5β-reductase (in liver only) and glucocorticoid receptor α (GRα) mRNA transcript levels by real time PCR. 30 mg of liver (or 100 mg adipose tissue) was homogenised and RNA extracted using Qiagen RNeasy Mini kits (West Sussex, UK), prior to quantification of RNA using the Ribogreen quantitation kit (Molecular Probes, Eugene, OR). RNA integrity was confirmed by agarose gel electrophoresis. cDNA was synthesized from 500 ng RNA using the Promega Reverse Transcription System (Madison, WI) and quantified using the Lightcycler 480 detection system (Roche, West Sussex, UK). A standard curve for each primer probe set was generated by serial dilutions (in nuclease-free water) of complementary DNA pooled from all rats. Samples were analysed in triplicate and the mean values of RNA abundance interpolated from the standard curve to calculate transcript levels. The mean of 18S and cyclophilin A was used as internal control to normalise abundances of transcript levels. The results are expressed as a proportion of the mean values in controls. Reverse transcriptase negative controls and RNA negative controls were used to confirm the absence of genomic DNA. Primers and probes for 18S, GRα, 5α-reductase type 1 and 5β-reductase were purchased pre-made from Applied Biosystems (Southampton, UK). Other primers/probes were custom ordered from Applied Biosystems using the following sequences; cyclophilin A (accession number NM_017101)–5′ CCC ACC GTG TTC TTC GAC AT 3′ (forward), 5′ GAA AGT TTT CTG CTG TCT TTG GAA CT 3′ (reverse), 5′ 6-FAM- CAA GGG CTC GCC ATC AGC CGT- TAMRA 3′ (probe); 11β-HSD1 (accession number NM_017080)–5′ TCA TAG ACA CAG AAA CAG CTT TGA AA 3′ (forward), 5′ CTC CAG GGC GCA TTC CT 3′ (reverse), 5′ 6-FAM- CTG GGA TAA TCT TGA GTC AAG CTG CTC CC- TAMRA 3′ (probe).

To assess 11β-HSD1 protein, 11β-HSD1 activity was measured in adipose tissue and liver in the dehydrogenase direction, as previously described [10], because dehydrogenase activity is more stable than reductase in vitro. Briefly, 500 µg/ml total protein of adipose tissue homogenate was incubated with 2 mM NADP, 0.2% glucose and 100 nM corticosterone (of which 10 nM 1,2,6,7-[^3^H]_4_-corticosterone (Amersham, Berkshire, UK)) at 37°C for 2 hours. Conversion to 1,2,6,7-[^3^H]_4_-11-dehydrocorticosterone was measured by HPLC with on line scintillation detection. For liver, the procedure was identical except 2 µg/ml total protein of liver homogenate was incubated with the above concentrations of NADP, glucose and corticosterone for 4 hours.

Hepatic 5β-reductase activity was measured in liver cytosol as previously described [11]. Briefly, 100 µg/ml protein was incubated in buffer (40 mM Na_2_HPO_4_, 1 mM dithiothreitol, 320 mM sucrose; pH 7.4), 2 mM NADPH and 2 µM corticosterone (of which 10 nM 1,2,6,7-[^3^H]_4_-corticosterone) at 37°C for 1 hour. Samples were analysed in duplicate and conversion to 1,2,6,7-[^3^H]_4_-5β-tetrahydrocorticosterone was measured by HPLC with on line scintillation detection. Hepatic 3α-hydroxysteroid dehydrogenase activity was measured in liver cytosol in an identical procedure to the 5β-reductase assay described above, using 2 µM 5α-dihydrotestosterone (of which 10 nM 1,2,4,5,6,7-[^3^H]_6_-5α-dihydrotestosterone) as the substrate while samples were incubated in duplicate at 37°C for 2 hours. Conversion to [^3^H]_6_-3α-5α,17β-androstanediol was measured by HPLC with online scintillation detection. 5α-Reductase activity was not measured due to instability of protein in vitro [22].

### Power Calculations and Statistical Analysis

Each dietary group comprised 12 rats, which provided a minimum of 85% power to detect to p<0.05 a difference of 15% between diets for the various mRNA transcripts at 30 weeks (8 weeks after any diet switch). Obese rats in the Western, low CHO and moderate CHO groups were compared with each other, while those in the Western group were also compared with lean rats on the control diet. All statistical analyses were performed using SPSS version 14. One way ANOVA with post-hoc testing using Fisher's least significant differences test was used to test for differences between both 2 diets (control vs Western) and 3 diets (Western, low CHO, moderate CHO). P<0.05 was considered significant. Data are presented as mean ± SEM.

## Results

### Body Composition and Biochemical Measurements

Body composition and biochemistry have been described in detail previously [21]. Briefly, the Western diet successfully induced an obese phenotype. After 22 weeks, rats fed the Western diet were heavier than the control-fed rats (649±5 vs 580±9 g, p<0.001), due to increased whole body fat mass (169±4 vs 86±6 g, p<0.001) with no significant difference in lean mass (444±3 vs 448±5 g).

Amongst obese rats, the low CHO group gained similar weight and fat mass to the Western group but lost lean mass, while those on the moderate CHO diet lost both fat and lean mass (Table 1). Lean rats on the control diet gained less weight than obese rats on the Western diet. Mean energy intakes were highest in rats fed low CHO, followed by those on Western, moderate CHO, and control diets (Table 1). Estimated energy expenditure was increased on the low CHO compared with the other three diets, and was increased on the moderate CHO diet compared with control (both p<0.05). In the OGTT at 28 weeks, fasting glucose was lower in the low CHO versus the Western and moderate CHO groups (Table 1). In addition, fasting insulin was lower on the low CHO and moderate CHO diets compared with the Western diet, while the total insulin area under the curve during the OGTT was lower on the moderate CHO compared with the Western diet. Fasting insulin and glucose were increased in the rats on the Western compared with the control diet (Table 1), as was total glucose area under the curve during the OGTT (p<0.001).

**Table 1:** Body composition, energy intake and OGTT results after 30 weeks.

	Control	Western	Low CHO	Moderate CHO
**Body weight (grams)**	590±9	676±5 ***	651±14	633±9 ^###^
**Fat mass (g)**	83±6	192±6 ***	189±7	150±6 ^### †††^
**Lean mass (g)**	456±5	445±5	425±8	441±7
**Delta weight (22–30 wks) (g)**	11±4	25±3 **	15±5	−25±6 ^### †††^
**Delta fat mass (22–30 wks) (g)**	−3±2	19±2 ***	21±3	−21±5 ^### †††^
**Delta lean mass (22–30 wks) (g)**	8±3	1±2	−8±3 ^#^	−9±3 ^#^
**Energy intakes (22–30 wks) (kcal/day)**	68±1	73±1 ***	83±2 ^###^	69±1 ^# †††^
**Fasting glucose (mmol/l)**	6.4±0.2	7.2±0.2 **	6.8±0.1 ^#^	7.3±0.2^†^
**Fasting insulin (mU/l)**	48±10	75±8 **	51±6 ^#^	48±3 ^##^
**OGTT Glucose AUC (mU/l per 2 h)**	960±17	1189±29 ***	1120±41	1163±48
**OGTT Insulin AUC (mU/l per 2 h)**	12173±2071	16443±1976	13070±1309	11359±1124 ^#^

Data presented as mean ± SEM for lean controls (n = 11), and diet-induced obese rats after 8 weeks on Western (n = 12), low carbohydrate (low CHO) (n = 12), and moderate carbohydrate (moderate CHO) diets (n = 12). Comparisons were made either between control and Western diets or between Western, low CHO and moderate CHO diets by one way ANOVA with post-hoc testing using Fisher's least significant differences test. ** p<0.01, *** p<0.001 Western vs control diet; ^#^ p<0.05, ^##^ p<0.01, ^###^ p<0.001 versus Western diet;^ †^ p<0.05, ^†††^ p<0.001 vs low CHO diet. **AUC** = area under curve. **OGTT** = oral glucose tolerance test.

Footnote: The difference between body weight and the sum of lean and fat tissue was attributed to skeletal mass and gut contents, both of which gave a low MRI signal.

### Effect of Obesity-Inducing Western Diet on Glucocorticoid Metabolism

**Figure 1:**
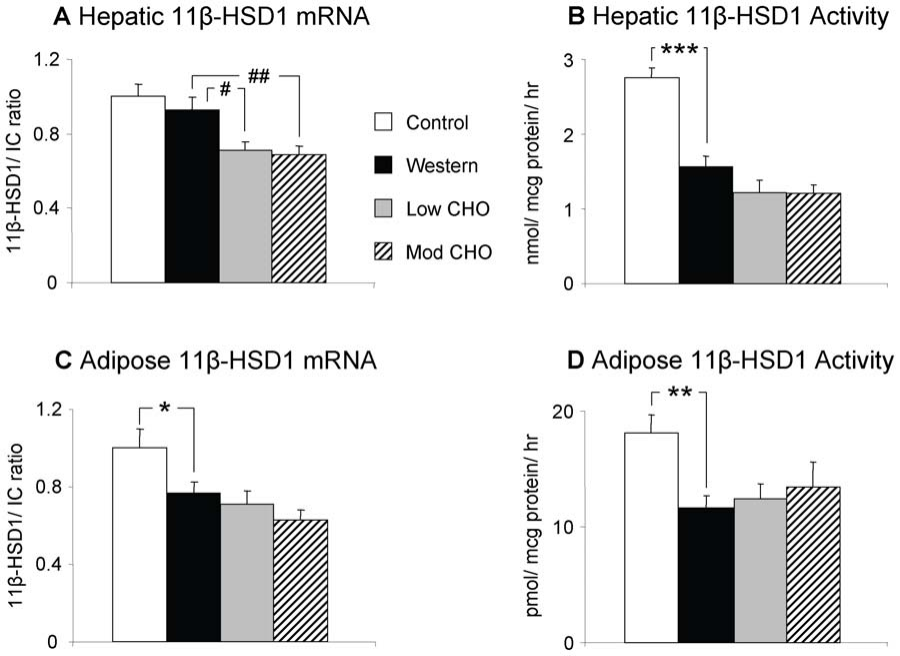
Hepatic and adipose tissue 11β-HSD1. Data presented as mean ± SEM for lean controls (n = 11, white columns), and diet-induced obese rats after 8 weeks on Western (n = 12, black columns), low carbohydrate (low CHO) (n = 12, grey columns), and moderate carbohydrate (mod CHO) diets (n = 12, striped columns). a) Hepatic 11β-HSD1mRNA levels. b) Hepatic 11β-HSD1 activity. c) Peri-renal adipose 11β-HSD1 mRNA levels. d) Peri-renal adipose 11β-HSD1 activity. The mean of 18S and cyclophilin A was used as internal control (IC) for mRNA data. mRNA transcript levels are expressed as a proportion of the mean values in controls. * p<0.05, ** p<0.01, *** p<0.001 Western vs control. # p<0.05, ## p<0.01 versus Western diet.

**Figure 2:**
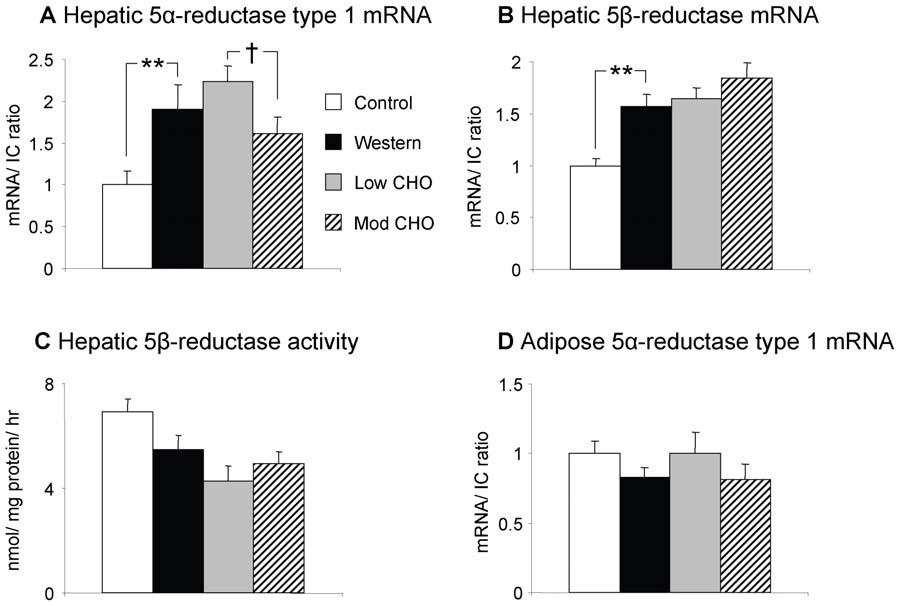
Hepatic and adipose tissue 5α-reductase type 1 and 5β-reductase mRNA and activity. Data presented as mean ± SEM for lean controls (n = 11, white columns), and diet-induced obese rats after 8 weeks on Western (n = 12, black columns), low carbohydrate (low CHO) (n = 12, grey columns), and moderate carbohydrate (mod CHO) diets (n = 12, striped columns). a) Hepatic 5α-reductase mRNA. b) Hepatic 5β-reductase mRNA. c) Hepatic 5β-reductase activity. d) Peri-renal adipose tissue 5α-reductase mRNA. The mean of 18S and cyclophilin A was used as internal control (IC). mRNA transcript levels are expressed as a proportion of the mean values in controls. ** p<0.01 Western vs control. † p<0.05 mod CHO vs low CHO.

**Figure 3:**
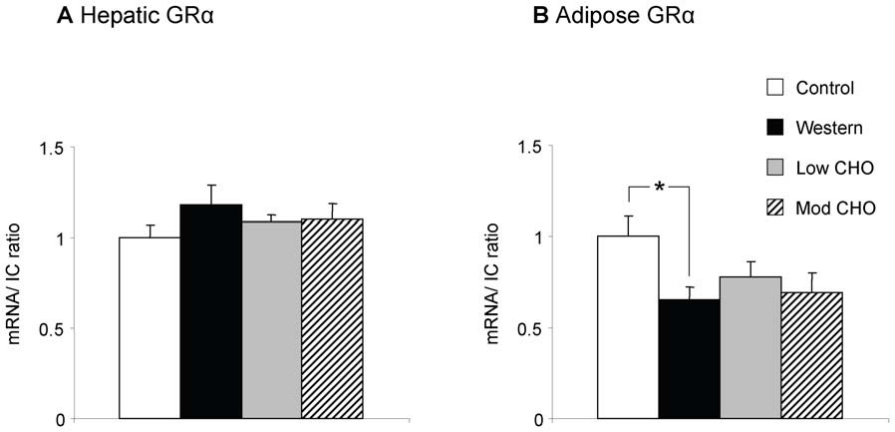
Hepatic and adipose tissue GRα mRNA. Data presented as mean ± SEM for lean controls (n = 11, white columns), and diet-induced obese rats after 8 weeks on Western (n = 12, black columns), low carbohydrate (low CHO) (n = 12, grey columns), and moderate carbohydrate (mod CHO) diets (n = 12, striped columns). a) Hepatic GRα mRNA. b) Peri-renal adipose tissue GRα mRNA. The mean of 18S and cyclophilin A was used as internal control (IC). mRNA transcript levels are expressed as a proportion of the mean values in controls. * p<0.05 Western vs control.

In the liver, 11β-HSD1 activity was significantly decreased on the Western diet compared with control, although 11β-HSD1 mRNA levels were unaltered (Figure 1a/b). Hepatic 5α-reductase type 1 and 5β-reductase mRNA were increased on the Western diet (Figure 2), although 5β-reductase activity was not different. Hepatic 3α-HSD activity was unaltered by the Western diet (data not shown). GRα mRNA was also unchanged between the two diets (Figure 3).

In peri-renal adipose tissue, both 11β-HSD1 mRNA and activity were decreased on the Western versus control diet (Figure 1c/d). GRα mRNA was also decreased on the Western diet (Figure 3). In contrast with the liver, 5α-reductase type 1 mRNA in adipose tissue was unaltered by the Western diet (Figure 2).

### Effect of Low and Moderate CHO Diets on Glucocorticoid Metabolism in Obese Rats

Hepatic 11β-HSD1 mRNA was reduced on both low CHO and moderate CHO diets compared with the Western diet with a similar trend in activity (both p<0.1) (Figure 1a/b). There was no difference in hepatic 11β-HSD1 mRNA or activity between the low and moderate CHO diets. Hepatic 5α-reductase type 1 mRNA and 5β-reductase mRNA and activity were unchanged for both the low CHO and moderate CHO diets compared with the Western diet, however 5α-reductase type 1 mRNA was increased on the low CHO versus the moderate CHO diet (Figure 2). 3α-HSD activity in the liver was unaltered between the low CHO, moderate CHO and Western diets (data not shown) as was GRα mRNA (Figure 3).

In adipose tissue, 11β-HSD1 mRNA and activity (Figure 1c/d), 5α-reductase type 1 (Figure 2) and GRα (Figure 3) mRNA were unaltered between the Western, low CHO and moderate CHO diets.

## Discussion

These data show that selective manipulations of dietary macronutrient content alter peripheral glucocorticoid metabolism in rats. The obesity-inducing ‘Western’ diet decreased liver 11β-HSD1 activity and increased 5α- and 5β-reductase mRNA compared with a low fat control diet, showing that a diet-induced obesity rodent model recapitulates the abnormal hepatic glucocorticoid metabolism observed in obesity in humans and Zucker rats [3], [4], [10], [11]. The decrease in visceral adipose tissue 11β-HSD1 mRNA and activity on the Western diet, however, contrasts with findings in obese humans [9], [23], [24], but is consistent with previous work in rodents showing that high fat diets decrease 11β-HSD1 in adipose tissue and liver [15], [16]. In addition, we have shown that GRα is regulated by dietary-induced obesity, as GRα mRNA expression in adipose tissue was decreased on the Western diet compared with lean controls, further reducing tissue glucocorticoid action.

Our dietary interventions closely paralleled those used in our previous human study, in which obese patients were studied during weight loss regimes using diets with varying CHO content [20]. In contrast with the human study, however, the low CHO diet did not increase hepatic 11β-HSD1 compared with the Western or moderate CHO diets; in fact, both low CHO and moderate CHO diets decreased hepatic 11β-HSD1 mRNA (with a trend for enzyme activity) compared with the Western diet, while there was no difference in direct comparison between the low CHO and moderate CHO diets. Body weight was significantly reduced in the rats on moderate CHO compared with low CHO and Western diets, suggesting that dietary macronutrient content regulates hepatic 11β-HSD1 independently of changes in body weight in rats. The lower carbohydrate content of the low CHO and moderate CHO diets may be the primary mediator of this reduction in hepatic 11β-HSD1, however the significantly increased protein content of both diets could also be responsible.

We have hypothesized that altered glucocorticoid metabolism in the liver in obese humans is secondary to hyperinsulinaemia [20] and can therefore be reversed by reducing carbohydrate intake. However this is not consistent with the current rodent data. Both low CHO and moderate CHO groups had lower fasting plasma insulin than those on the Western diet (presented in detail in [21]), but hepatic 11β-HSD1 decreased further rather than increased in these groups. In addition, hepatic 5α-reductase type 1 mRNA was increased on the low CHO compared with the moderate CHO diet, in contrast with our observations in humans. This is probably secondary to the rats losing weight on the moderate CHO diet while those on the low CHO gained weight. Obesity is associated with increased 5α-reductase activity in humans [5], while we [20], [25] and others [26] have previously shown that weight loss reduces 5α-reductase activity. In contrast, 5β-reductase was unaffected by dietary manipulation in obese rats.

In visceral adipose tissue, glucocorticoid metabolism and GRα were unaltered between the Western, low CHO and moderate CHO diets, which is consistent with humans [20]. While the Western diet (with 37% fat) decreased 11β-HSD1 in adipose tissue compared with control (9% fat), increasing the fat content further from 37% to 56% (on low CHO) did not further alter adipose 11β-HSD1, indicating there may be a threshold effect of dietary fat content. In addition, rats on the moderate CHO had significant weight loss without altering 11β-HSD1, indicating that dietary fat regulation of adipose 11β-HSD1 is independent of both protein and carbohydrate content, and also of changes in weight. Only peri-renal adipose tissue was studied, so we do not know if dietary regulation of 11β-HSD1 was similar in other adipose sites, although dietary regulation of 11β-HSD1 has been observed across all studied fat depots in mice [16].

Whilst the current data suggest important species differences may exist that should be considered when extrapolating from rodent to human glucocorticoid metabolism, some potential confounders may help to explain the differences. For example, energy intake differed between all four diets, in part because the diets were not energy isodense. The macronutrient content of these diets was designed to closely mirror those used in the human study, however the main component of dietary fat (maize oil) had a lower saturated fat and higher mono-unsaturated and poly-unsaturated content than the human diets; it is possible that the type of dietary fat is important in regulating glucocorticoid metabolism. Weight gain was also different between diets, particularly on the moderate CHO diet when the rats lost weight. The reasons for weight loss in the moderate CHO group are unclear, but their energy intakes were significantly lower than the other obese groups and in fact were similar to those of the lean control group. Although 11β-HSD1 mRNA and activity closely correlated in adipose tissue, this was not the case in liver with the Western diet. This lack of correlation between transcript levels and activity has been observed previously in the liver and other tissues [15], [24], [27]. We also observed dissociated changes of 5β-reductase mRNA and enzyme activity.

The mechanism through which dietary fat content decreases hepatic and adipose tissue glucocorticoid regeneration is unknown. Possible candidates are the peroxisome proliferator activated receptors (PPARs), as dietary fats are the endogenous ligands for PPARs. PPARα-agonists decrease 11β-HSD1 in the liver in mice [28], while PPARγ-agonists decrease 11β-HSD1 in rodent adipose tissue [29], [30]. Interestingly, these effects are not acutely observed in humans [31], which could potentially account for the differences in dietary regulation observed between species. The liver X receptor (LXR) is another potential mediator of these effects, as LXR decreases 11β-HSD1 in the liver and adipose tissue [32], [33]. Further work is required to fully understand these mechanisms.

To conclude, we have shown that diet-induced obesity in rats increases hepatic glucocorticoid metabolism by reducing 11β-HSD1 and increasing 5α- and 5β-reductase expression, recapitulating the abnormal metabolism observed in human obesity. However, unlike in humans, a low carbohydrate diet does not increase hepatic 11β-HSD1 in rats, indeed lower carbohydrate content may decrease hepatic 11β-HSD1 further. Confirming previous results in rodents, high dietary fat content reduces glucocorticoid signalling by decreasing 11β-HSD1, and also GRα, in adipose tissue. Conversely, hepatic 5α-reductase is increased in association with increases in weight. Manipulation of dietary composition may chronically regulate 11β-HSD1 resulting in altered local glucocorticoid concentrations, which may modify the metabolic efficacy of various dietary strategies.
